# Molecular Mechanisms for Regulating Postnatal Ductus Arteriosus Closure

**DOI:** 10.3390/ijms19071861

**Published:** 2018-06-25

**Authors:** Yu-Chi Hung, Jwu-Lai Yeh, Jong-Hau Hsu

**Affiliations:** 1 Graduate Institute of Medicine, College of Medicine, Kaohsiung Medical University, Kaohsiung 807, Taiwan; u8901035@gmail.com (Y.-C.H.); jwulai@kmu.edu.tw (J.-L.Y.); 2 Department of Pediatrics, St. Joseph Hospital, Kaohsiung 807, Taiwan; 3 Department of Pharmacology, College of Medicine, Kaohsiung Medical University, Kaohsiung 807, Taiwan; 4 Department of Medical Research, Kaohsiung Medical University Hospital, Kaohsiung 807, Taiwan; 5 Department of Marine Biotechnology and Resources, National Sun Yat-sen University, Kaohsiung 804, Taiwan; 6 Department of Pediatrics, Kaohsiung Medical University Hospital, Kaohsiung Medical University, Kaohsiung 807, Taiwan; 7 Department of Pediatrics, Faculty of Medicine, College of Medicine, Kaohsiung Medical University, Kaohsiung 807, Taiwan

**Keywords:** ductus arteriosus, endothelial cells, extracellular matrix, smooth muscle cells, vascular remodeling

## Abstract

The ductus arteriosus (DA) connects the main pulmonary artery and the aorta in fetal circulation and closes spontaneously within days after birth in normal infants. Abnormal patent DA (PDA) causes morbidities and mortality, especially in preterm infants. Closure of the DA is a complex interactive process involving two events: functional and anatomic closure. Functional closure by smooth muscle contraction was achieved through the regulatory factors of vaso-reactivity. These factors include oxygen sensing system, glutamate, osmolality, prostaglandin E_2_, nitric oxide, and carbon monoxide. Anatomic closure by vascular remodeling involved several vascular components including endothelium, extracellular matrix, smooth muscle cells, and intraluminal blood cells. Despite advances in understanding of PDA pathogenesis, the molecular mechanism for regulation of DA closure is complex and not fully understood. In this article we review recent evidence regarding the molecular mechanisms of DA closure.

## 1. Introduction

The ductus arteriosus (DA) is a vital vessel that connects pulmonary circulation and systemic circulation in the fetus. Closure of the DA is mostly completed within three days of life in healthy term newborns. The incidence of an isolated patent DA (PDA) ranges from 3 to 8 per 10,000 live births among term infants [1] and is estimated at up to 30 percent in very low birth weight infants (birth weight below 1500 g) [2]. The PDA is a hemodynamic burden in preterm infants and is also the leading cause of mortality and morbidity among these infants [3]. However, maintaining the patency of the DA is life-saving in infants with ductus-dependent congenital heart diseases. Therefore, proper manipulation of DA patency is essential in neonatal intensive care and investigation of its molecular mechanisms is an important field in vascular biology and pediatrics.

Generally, DA closure involves two phases: functional and anatomical closure. Functional closure occurring within hours after birth is caused by DA constriction and the following anatomical closure is mediated mainly by vascular remodeling. After birth, increased oxygen tension and declined prostaglandin E_2_ (PGE_2_) are two major factors for DA constriction [4]. Subsequent DA remodeling is associated with several histological changes: internal elastic lamina (IEL) disruption, lifting and ingrowth of endothelial cells (ECs), subendothelial edema due to deposition of extracellular matrix (ECM), migration and proliferation of the SMCs into the subendothelial space [5,6,7]. These histological changes result in intimal cushion for permanent closure of the DA. In this article, we review both mechanisms of functional and anatomical closure of the DA.

## 2. Functional Closure

**Figure 1:**
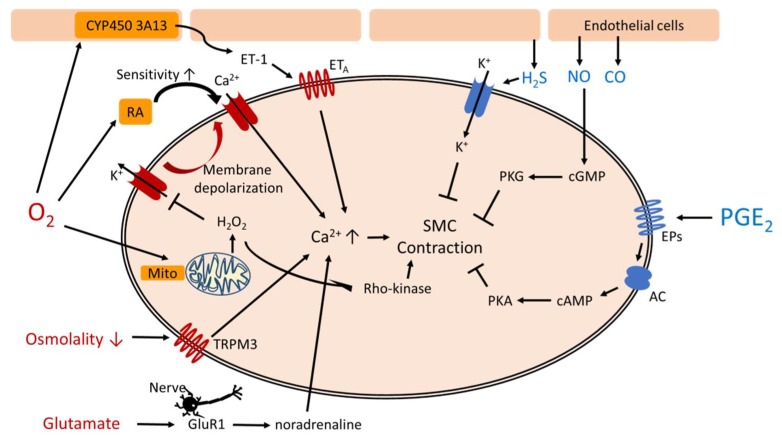
Pathways mediating functional closure of the ductus arteriosus. AC: adenylyl cyclase, cAMP: cyclic adenosine monophosphate, cGMP: cyclic guanosine monophosphate, EPs: PGE_2_ receptors, ET: endothelin, GluR1: glutamate inotropic receptor subunit 1, Mito: mitochondria, PGE_2_: prostaglandin E_2_, PKA: protein kinase A, PKG: protein kinase G, RA: retinoic acid, SMC: smooth muscle cells, TRPM3: transient receptor potential melastatin 3.

During fetal life, intrauterine hypoxia works synergistically with high circulating PGE_2_ to maintain DA patency. After birth, the DA constricts in response to elevated oxygen tension and declined PGE_2_ level [8,9]. However, the preterm infants often have hypoxic events such as respiratory distress syndrome or bronchopulmonary dysplasia, resulting higher incidence of PDA. There are many factors controlling the DA vascular tone (Table 1). Figure 1 shows complex pathways mediating functional closure of the DA.

### 2.1. Vasoconstriction

#### 2.1.1. Oxygen Pathways

Several mechanisms were found recently to underlie the vasoconstrictive response of high oxygen tension in DA. Archer et al. demonstrated that DA smooth muscle cells (DASMCs) can sense oxygen via dynamic mitochondrial network [35]. They showed O_2_-induced DA constriction was initiated by inhibition of a voltage-gated potassium channel, which caused membrane depolarization, activation of L-type calcium channels and increment in intracellular calcium (Ca^2+^) [14]. H_2_O_2_ produced by mitochondrial electron transport chain complex served as an oxygen mediator to inhibit potassium channels [15]. Through mitochondrial fission, elevated oxygen tension increased reactive oxygen species (ROS) levels and mitochondrial complex I activity [16]. In brief, oxygen-induced increment of ROS (e.g., H_2_O_2_) inhibits potassium channel and subsequent membrane depolarization causes Ca^2+^ influx due to opening of calcium, inducing DASMCs contraction.

Recent evidence demonstrates that the role of Rho-kinase pathway to sustain DA constriction via the mitochondrial system. The oxygen-induced increment of mitochondrial ROS activates the Rho-kinase pathway and induces RhoB and Rho-associated protein kinase-1 expression in human and rabbit DA [17]. The Rho-kinase pathway promotes phosphorylation of myosin phosphatase targeting protein and this phosphorylation inhibits myosin light chain phosphatase, thereby increasing the phosphorylation and activity of the myosin light chain, which leads to DASMC contraction. The activation of the Rho-kinase pathway thus induces calcium sensitization, which sustains DA constriction through a positive feedback mechanism.

There is some evidence suggesting that cytochrome P_450_ (CYP450) and endothelin-1 (ET-1) also jointly participate in the mechanisms underlying oxygen-induced DA constriction. The level of ET-1 increased in response to oxygen and acted as DA constrictor via ET_A_ receptor [36,37,38]. The CYP450-based mechanism mediates the constrictive response of the DA to oxygen, possibly by stimulating the release and synthesis of ET-1 [21,22].

Another possible oxygen sensing factor is retinoic acid, a metabolite of vitamin A. Wu et al. found that fetal rats born from maternally vitamin A-treated group had better DA contraction induced by oxygen but not by KCl [24]. Yokoyama et al. showed that maternally administered vitamin A significantly upregulated the expression levels of α_1G_ subunit of voltage-dependent calcium channel, which is activated by oxygen-induced inhibition of potassium channel [25].

#### 2.1.2. Pathways Unrelated to Oxygen

Glutamate, an amino acid, has been recently found to promote DA contraction through glutamate inotropic receptor subunit 1 (GluR1)-mediated noradrenaline production. Fujita et al. showed that glutamate increased noradrenaline production in the rat DA and subsequent glutamate-induced DA contraction was attenuated by the GluR receptor antagonist or the adrenergic receptor α_1_ blocker [28]. This evidence suggests that nutritional adjustment with supply amino acid may have therapeutic implications in newborn infants with PDA.

Recent evidence suggests that hypo-osmolality has a role in mediating DA constriction. This mechanism is mediated by regulating Ca^2+^, potentially through the transient receptor potential melastatin 3 (TRPM3) pathway. Aoki et al. found that in rats that hypo-osmotic sensor TRPM3 was more upregulated in the DA than in the aorta [31]. They also demonstrated that rats experienced transient hypo-osmolality after birth, which contributed to rat DA constriction.

There are other agents circulating in the blood conveying vasoconstrictive effects on DA. For example, bradykinin shows biphasic effect at rabbit DA through two different receptors, BK-1 and BK-2 receptors. As bradykinin concentration increases, DA has predominantly constrictive responses through BK-1 receptor [32]. Corticosteroids also induce DA constriction, in combination with indomethacin, probably through attenuating the sensitivity of the DA to PGE_2_ [33,34].

### 2.2. Vasodilation

#### 2.2.1. PGE_2_

In current clinical settings, PGE_1_ administration is the only medical treatment for maintaining DA patency in neonates with DA-dependent congenital heart diseases [39]. PGE_2_ is produced in both the placenta and the DA in fetal circulation. It maintains DA patency through various PGE receptors (EP_2_, EP_3_, and EP_4_) [10,11,12]. Activation of PGE_2_ receptors increases intracellular cyclic AMP (cAMP) via adenylyl cyclases and the increased cAMP level inhibits myosin light chain kinase, subsequently dilating DA [11,13]. After birth, the PGE_2_ level declines due to pulmonary catabolism of PGE_2_ and the removal of the placenta [9,10]. PGE_2_ receptors (EP_3_ and EP_4_) also decrease in numbers after birth [11]. Postnatal decline of PGE_2_ signaling has been postulated to be the fundamental mechanism for DA closure [40]. Moreover, elevated oxygen tension can downregulate DA sensitivity to PGE_2_, thus attenuating the postnatal vasodilating response [41,42].

#### 2.2.2. Vasodilating Factors Unrelated to PGE

Nitric oxide (NO) has been shown to be a vasodilator in DA. NO is produced by endothelial nitric oxide synthase (eNOS) in ECs and then diffuses into adjacent SMCs to bind with soluble guanylyl cyclase (sGC). The activated sGC causes production of cyclic guanosine monophosphate (cGMP), which decreases Ca^2+^. The lowering Ca^2+^ relaxes the SMCs and promotes vasodilation [43]. In DA, NO can be synthesized by eNOS in the endothelium of DA lumen and vasa vasorum [18]. Indeed, combined use of indomethacin and NOS inhibitor was shown to have more potent constricting efficacy in DA than indomethacin alone in premature baboons [19]. Intriguingly, indomethacin was also found to promote vasodilatory function of NO in mouse DA [20]. These paradoxical effects could explain the failure of indomethacin therapy in about 30% of premature neonates in clinical practice [44].

Similar with NO, another vasodilating pathway mediated through cGMP is the family of natriuretic peptides. They are cardiac-producing peptides that can dilate vessels through the particulate GC-cGMP pathway [45]. Atrial natriuretic peptide has been shown to dilate rat DA in vivo [23]. Our recent study demonstrated that higher B-type natriuretic peptides (BNP) convey anti-remodeling effects in the pulmonary artery SMCs [46]. Furthermore, in the setting of neonatal intensive care unit, plasma levels of BNP are associated with poor response to indomethacin treatment in preterm infants with PDA [44]. Taken together, this evidence suggests the role of BNP in DA control, but this warrants further investigation.

In addition to NO, other gases including carbon monoxide (CO) and hydrogen sulfide (H_2_S) can also dilate DA. The CO-forming enzyme, heme oxygenase-1 and -2, identified in DA tissue, was shown to produce CO in the DASMCs [26]. CO dilates DA due to inhibition of a CYP450-based monooxygenase reaction conditioning the formation of the ET-1 [27]. Recently, Baragatti et al. demonstrated H_2_S synthetic enzyme in the mice DA and confirmed the H_2_S-induced vasodilatory effects of DA [29]. Interestingly, H_2_S was found to have biphasic effects, inducing vasoconstriction at lower concentrations while causing vasodilation at higher concentrations [30]. However, in the chicken DA, the vasodilatory effect was not shown [47]. The inter-species differences and dose-specific vasoreactive mechanisms of H_2_S in DA are not fully understood and warrant further investigations.

## 3. Anatomical Closure

**Figure 2:**
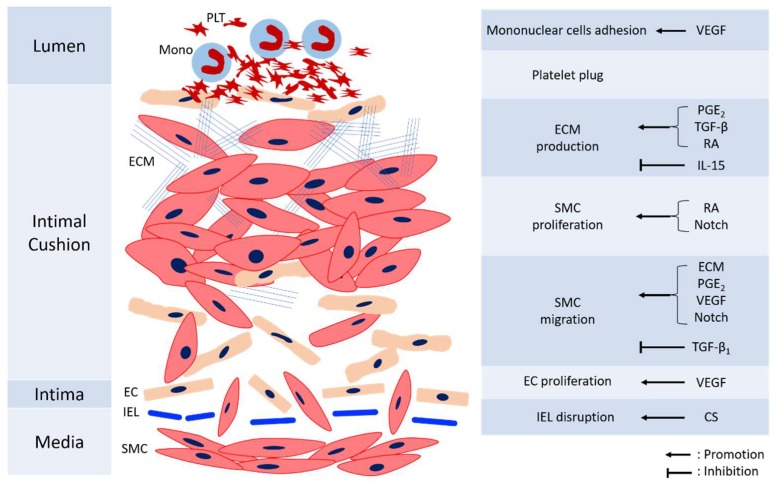
The diagram of anatomical closure of the ductus arteriosus. CS: chondroitin sulfate, EC: endothelial cells, ECM: extracellular matrix, IEL: internal elastic laminae, IL-15: Interleukin-15, Mono: monocyte, PDGF: platelet-derived growth factor, PGE_2_: prostaglandin E_2_, PLT: platelet, RA: retinoic acid, SMC: smooth muscle cells, TGF-β: transforming growth factor-β, VEGF: vascular endothelial growth factor.

Remodeling of DA is essential to permanent anatomical closure to prevent re-opening. The process is complex and not fully understood, with several mechanisms including intimal cushion formation, SMC migration and proliferation, ECM production, EC proliferation, and blood cell interaction. These steps interact with each other and construct an orchestrated process. Figure 2 shows the mechanisms involved in the anatomic closure of the DA. The detailed references of mechanisms mediating various cells for anatomical closure are shown in Table 2.

### 3.1. Factors Regulating SMC Proliferation and Migration

Similar to the vascular remodeling of other diseases such as atherosclerosis and pulmonary hypertension, SMC migration and proliferation play important roles in DA remodeling. DA remodeling starts with separation of the EC from the IEL resulting in creation of subendothelial space for migration and proliferation of undifferentiated SMCs [64]. These factors include PGE2, retinoic acid, transforming growth factor-β_1_ (TGF-β_1_), and Notch signaling.

#### 3.1.1. PGE_2_

PGE_2_ induces DASMC migration through exchange protein activated by cAMP (Epac) pathway. Epac signaling is also regulated by cAMP but is distinctly different from the PKA pathway [65]. Serial activations of the PGE_2_-EP4-cAMP-Epac signaling pathway induce DASMC migration without changing SMC proliferation and hyaluronan production [48]. PGE_2_ has paradoxical effects on the functional and anatomical aspects of DA closure, that is, vasodilatation and remodeling.

#### 3.1.2. Retinoic Acid

Retinoic acid also participates in vascular remodeling via promoting SMC and ECM proliferation. Wu et al. showed that retinoic acid stimulated the growth of DASMCs by the stimulation of proliferating cell nuclear antigen expression and decreased apoptosis [54]. Yokoyama et al. demonstrated that maternally administrated vitamin A increased the production of fibronectin and hyaluronic acid, promoting intimal thickening in the DA at preterm rats [55]. Taken together, retinoic acid mediates both vasoconstriction and vascular remodeling.

#### 3.1.3. TGF-β_1_

TGF-β_1_ anchors the SMC’s cytoskeleton to the ECM, making SMCs more adherent to ECM and less migrative. TGF-β_1_ increases focal plaque formation in DASMCs by increasing adhesion of the integrin with the cytoskeleton, possibly maintaining the tension necessary to sustain DA contracture during remodeling [49,66].

#### 3.1.4. Notch Signaling

The Notch system is highly expressed in human vasculature and regulates cell behavior, including proliferation, migration, and angiogenesis [67]. Recent reports suggest that it has a role in DA remodeling. Baeten et al. showed that the loss of Notch receptors in DASMCs is associated with downregulated contractile SMC gene expression, contributing to the formation of PDA [68]. Krebs et al. demonstrated that Notch signaling is required for contractile smooth muscle cell differentiation and DA closure in mice [69]. Our recent study suggests a role of Notch signaling in the proliferation and migration of DASMCs [50]. Specifically, we found that γ-secretase inhibitor DAPT, a Notch signaling inhibitor, could prevent the angiotensin II-induced proliferation and migration of DASMCs. These effects are potentially mediated by attenuated calcium overload, reduced ROS production, and deactivations of ERK1/2, JNK, and Akt signal transduction through the Notch3-HES1/2/5 pathway.

### 3.2. Extracellular Matrix (ECM)

It has been found in many vascular proliferative diseases that the ECM can promote SMC migration and proliferation [70]. ECM consists of hyaluronan, fibronectin, chondroitin sulfate, and elastin, and each of them has a different role in DA remodeling.

#### 3.2.1. Hyaluronan

Hyaluronan is important during DA remodeling due to its effects on promoting DASMC migration. It is regulated by other factors, including TGF-β, PGE_2_, and interleukin-15 (IL-15). TGF-β is produced in ECs and can promote synthesis of hyaluronan and chondroitin sulfate in DA [56]. PGE_2_ regulates hyaluronan production via the EP4-cAMP-PKA signaling pathway [71]. PGE_2_-mediated activation of EP4 leads to increased cAMP production and PKA signaling, leading to increased hyaluronan synthase activity in DASMCs. Chronic EP4 can also stimulate hyaluronan production [51]. IL-15 is predominantly expressed in the IEL in rat DA and inhibits hyaluronan production [57].

#### 3.2.2. Fibronectin

Fibronectin is secreted by DASMCs and can promote SMC migration into the subendothelium in the process of intimal cushion formation. DA patency can be maintained by inhibiting fibronectin-dependent intimal cushion formation [52]. In addition, maternally administrated vitamin A increased fibronectin production in the DA of neonatal rats [55].

#### 3.2.3. Chondroitin Sulfate

Chondroitin sulfate promotes DA remodeling through supporting the stability of hyaluronan and impairing the assembly of elastin fibers. Chondroitin sulfate causes 67-kD elastin binding protein to be released from the SMC surface, impairing elastin assembly [62]. Chondroitin sulfate promotes SMC migration indirectly by promoting detachment of SMCs from elastin and upregulates synthesis of fibronectin, which facilitates migration of SMCs through IEL [72].

#### 3.2.4. Elastin

Elastin confers elasticity of blood vessels and contributes to maintaining PA patency. The loss of elastin-binding protein and the production of elastin peptides can enhance DASMC migration [73]. Intriguingly, the production of elastin is regulated by PGE_2_ and oxygen. A recent study indicates that PGE_2_ can inhibit elastogenesis via the EP4 receptor and such attenuated elastin formation promotes vascular collapse and subsequent DA closure after birth [58]. In addition, oxygenation reduces elastin secretion in DASMCs [59]. Thus, from the perspective of elastogenesis, both PGE2 and oxygen play a role in anti-remodeling.

### 3.3. Factors Affecting Endothelial Cells (ECs)

In the process of DA remodeling, ECs separates from IEL to create a subendothelial space for the further migration of SMCs and ECs. The migration of ECs is influenced by integrins and VEGF.

Integrins are transmembrane receptors that create traction with surrounding ECM and provide signals for cytoskeleton rearrangement and initiation of cytoplasmic flow. Both ECs and SMCs experience an increase in their integrin supply during intimal cushion formation. Indeed, preterm infants with PDA were found to have downregulation of integrin expression [74]. Thus, integrin may participate in the interaction between ECM and ECs during DA remodeling, as it does between ECM and SMCs [75].

Vascular endothelial cell growth factor (VEGF) is a hypoxia-induced growth factor and can stimulate EC proliferation and migration. VEGF regulates DA remodeling by stimulating EC proliferation and SMC migration and is induced by tissue hypoxia [53]. Clyman et al. proposed an important role of VEGF in DA remodeling [60]. They demonstrated that initial functional vasoconstriction causes a loss of luminal blood flow, producing a hypoxic zone in the DA muscle media layer. They also found that distribution of VEGF is closely associated with the area of hypoxia in the constricting DA. In addition, anti-VEGF antibody was found to inhibit mononuclear cells from adhering to the DA lumen and decreases intimal cushion expansion [61].

### 3.4. Blood Cells’ Interaction

Circulating blood cells adhering to DA lumen have essential roles during DA remodeling. Mononuclear cells activated by inflammatory responses-induced vascular wall ischemia have recently been postulated as necessary for DA remodeling. After DA constriction, VLA4^+^ mononuclear cells (monocytes and macrophages) adhere to the ductus lumen via vascular cell adhesion molecule-1 expressed in the luminal cells [76]. The degree of mononuclear cells adhesion is correlated with the extent of intimal cushion formation [61].

Platelets also have a central role in permanent DA closure. Echtler et al. demonstrated that, during DA constriction, ECs become detached and trigger the recruitment of platelets passing through the constricted DA [77]. The formation of a platelet plug seals the residual lumen of the constricted DA and facilitates luminal remodeling. Engur et al. reported that platelet-derived growth factor levels were lower in infants who had persistent PDA after birth [63]. Emerging evidence shows the relationships between thrombocytopenia and the failure of spontaneous closure of DA [78,79].

## 4. Pharmacological Agents for Management of DA Patency

Current medications for the management of DA patency mainly convey physiological effects on vascular tone by vasodilation or vasoconstriction, rather than a remodeling effect. Table 3 summarizes the currently used or experimental agents to close or open DA.

### 4.1. Agents for Closing the DA

Drugs for closing the DA involve inhibition of prostaglandin (PG) production. In current clinical settings, indomethacin or ibuprofen is administrated for closing the DA in preterm newborn with DA-induced heart failure. Indomethacin and ibuprofen inhibit cyclooxygenase-1 and cyclooxygenase-2, which convert arachidonic acid to PGG_2_ for further production of various PGs. Among the PGs, PGE_2_ is the most potent vasodilator to open DA [91]. Indomethacin and ibuprofen both inhibit PGE_2_ production and are effective in closing the PDA in preterm infants. Oral ibuprofen may be the preferred agent due to feasibility and fewer side effects [80]. Recently, acetaminophen has been found to achieve DA closure in preterm infants [82]. Acetaminophen reduces PG production, probably through affecting peroxidase segment of cyclooxygenase [83]. Although some studies showed that acetaminophen was as effective as ibuprofen in closing the PDA [84,85], conflicting results preclude the routine use of acetaminophen for closing the PDA so far [86]. Further clinical studies are needed to reveal the efficacy of acetaminophen in closing the PDA.

### 4.2. Agents for Opening the DA

Several agents are found to maintain DA patency through conveying vasodilatory effect in clinical practice or animal studies. Clinically, PGE_1_ (Alprostadil) is administered in infants with ductus-dependent congenital heart diseases to maintain DA patency. PGE_1_ binds to the EP4 receptor and then increases intracellular cAMP levels, which inhibit myosin light-chain kinase, resulting in the relaxation of the DA [81]. Milrinone, a phosphodiesterase 3 inhibitor, can dilate the rat DA through increasing cAMP levels [87]. Enalapril, an angiotensin-converting enzyme inhibitor, can also delay DA closure when given during caesarean delivery and can reopen the closed DA temporarily when given at 180 min of life in newborn rats [88]. A nonselective endothelin receptor antagonist (ERA), TAK-044, was found to inhibit DA construction in rats [89]. NO regulates the patency of the DA through the NO-cGMP pathway [90]. We have recently found that inhibition of the Notch pathway may convey anti-remodeling effects on DASMCs, suggesting its potential role in DA control [50]. Our ongoing study shows that BNP, an activator of PKG-cGMP, can prevent postnatal DA closure. Therefore, the cGMP pathway may be a potential research target in regulating DA patency.

## 5. Conclusions

DA closure consists of complex interactive processes involving vascular tone and vascular remodeling. The current clinical pharmacological strategy of regulating DA is based on the PG pathway and has some adverse effects and limitations. However, many other agents with vasodilatory or anti-remodeling effects through non-PG pathways have been shown with potential roles in maintaining DA patency, such as the NO, BNP, or Notch pathways. However, these agents require more animal or clinical studies to confirm their efficacy. Therefore, future research targeting a new pharmacological strategy of DA is essential in the fields of vascular biology and pediatrics.

## Figures and Tables

**Table 1:** Factors mediating functional closure of the ductus arteriosus.

Vasoconstrictors	References	Vasodilators	References
Oxygen sensing		Prostaglandin E_2_	[10,11,12,13]
Mitochondria	[14,15,16,17]	Nitric oxide	[18,19,20]
Cytochrome P_450_	[21,22]	Natriuretic peptides	[23]
Retinoic acid	[24,25]	Carbon monoxide	[26,27]
Glutamate	[28]	Hydrogen sulfide	[29,30]
Hypoosmolality	[31]		
Bradykinin	[32]		
Corticosteroid	[33,34]		

**Table 2:** Factors mediating anatomical closure of the ductus arteriosus.

Cells	Mechanisms	Factors	Effects	Reference
SMCs	Migration	PGE_2_	+	[48]
		TGF-β_1_	−	[49]
		Notch	+	[50]
		Fibronectin & Hyaluronan	+	[51,52]
		VEGF	+	[53]
	Proliferation	Retinoic acid	+	[54]
		Notch	+	[50]
ECM production	Hyaluronan	Retinoic acid	+	[55]
		TGF-β	+	[56]
		PGE_2_	+	[51]
		IL-15	−	[57]
	Fibronectin	Retinoic acid	+	[55]
	Chondroitin sulfate	TGF-β	+	[56]
	Elastin	PGE_2_	−	[58]
		Oxygen	−	[59]
ECs	Proliferation	VEGF	+	[53,60,61]
IEL	Disruption	Chondroitin sulfate	+	[62]
Blood cells	Mononuclear cells adhesion	VEGF	+	[61]
	Platelet plug	PDGF	+	[63]

ECs: endothelial cells, ECM: extracellular matrix, IEL: internal elastic laminae, IL-15: Interleukin-15, PDGF: platelet-derived growth factor, PGE_2_: prostaglandin E_2_, SMCs: smooth muscle cells, TGF-β: transforming growth factor-β, VEGF: vascular endothelial growth factor.

**Table 3:** Clinical and experimental agents for management of ductus arteriosus.

Ductus Closure	References	Ductus Patency	References
Indomethacin *	[80]	Notch inhibitor	[50]
Ibuprofen *	[80]	Prostaglandin E_1_ *	[81]
Acetaminophen	[82,83,84,85,86]	Milrinone	[87]
		Enalapril	[88]
		Endothelin receptor antagonist	[89]
		Nitric oxide	[90]

* Currently used drugs in patients.
